# Development of a Large-Scale Dataset of Chest Computed Tomography Reports in Japanese and a High-Performance Finding Classification Model: Dataset Development and Validation Study

**DOI:** 10.2196/71137

**Published:** 2025-08-28

**Authors:** Yosuke Yamagishi, Yuta Nakamura, Tomohiro Kikuchi, Yuki Sonoda, Hiroshi Hirakawa, Shintaro Kano, Satoshi Nakamura, Shouhei Hanaoka, Takeharu Yoshikawa, Osamu Abe

**Affiliations:** 1 Division of Radiology and Biomedical Engineering, Graduate School of Medicine, The University of Tokyo, 7-3-1 Hongo, Bunkyo-ku, Tokyo, 113-8655, Japan, 81 3-3815-5411; 2 Department of Computational Diagnostic Radiology and Preventive Medicine, The University of Tokyo Hospital, Tokyo, Japan; 3 Department of Radiology, School of Medicine, Jichi Medical University, Shimotsuke, Japan; 4 Department of Diagnostic Radiology, Toranomon Hospital, Tokyo, Japan

**Keywords:** radiology reports, machine translation, structured findings, medical language models, computed tomography, multilingual datasets, radiology report classification

## Abstract

**Background:**

Recent advances in large language models have highlighted the need for high-quality multilingual medical datasets. Although Japan is a global leader in computed tomography (CT) scanner deployment and use, the absence of large-scale Japanese radiology datasets has hindered the development of specialized language models for medical imaging analysis. Despite the emergence of multilingual models and language-specific adaptations, the development of Japanese-specific medical language models has been constrained by a lack of comprehensive datasets, particularly in radiology.

**Objective:**

This study aims to address this critical gap in Japanese medical natural language processing resources, for which a comprehensive Japanese CT report dataset was developed through machine translation, to establish a specialized language model for structured classification. In addition, a rigorously validated evaluation dataset was created through expert radiologist refinement to ensure a reliable assessment of model performance.

**Methods:**

We translated the CT-RATE dataset (24,283 CT reports from 21,304 patients) into Japanese using GPT-4o mini. The training dataset consisted of 22,778 machine-translated reports, and the validation dataset included 150 reports carefully revised by radiologists. We developed CT-BERT-JPN, a specialized Bidirectional Encoder Representations from Transformers (BERT) model for Japanese radiology text, based on the “tohoku-nlp/bert-base-japanese-v3” architecture, to extract 18 structured findings from reports. Translation quality was assessed with Bilingual Evaluation Understudy (BLEU) and Recall-Oriented Understudy for Gisting Evaluation (ROUGE) scores and further evaluated by radiologists in a dedicated human-in-the-loop experiment. In that experiment, each of a randomly selected subset of reports was independently reviewed by 2 radiologists—1 senior (postgraduate year [PGY] 6‐11) and 1 junior (PGY 4‐5)—using a 5-point Likert scale to rate: (1) grammatical correctness, (2) medical terminology accuracy, and (3) overall readability. Inter-rater reliability was measured via quadratic weighted kappa (QWK). Model performance was benchmarked against GPT-4o using accuracy, precision, recall, *F*_1_-score, ROC (receiver operating characteristic)—AUC (area under the curve), and average precision.

**Results:**

General text structure was preserved (BLEU: 0.731 findings, 0.690 impression; ROUGE: 0.770‐0.876 findings, 0.748‐0.857 impression), though expert review identified 3 categories of necessary refinements—contextual adjustment of technical terms, completion of incomplete translations, and localization of Japanese medical terminology. The radiologist-revised translations scored significantly higher than raw machine translations across all dimensions, and all improvements were statistically significant (*P*<.001). CT-BERT-JPN outperformed GPT-4o on 11 of 18 findings (61%), achieving perfect *F*_1_-scores for 4 conditions and *F*_1_-score >0.95 for 14 conditions, despite varied sample sizes (7‐82 cases).

**Conclusions:**

Our study established a robust Japanese CT report dataset and demonstrated the effectiveness of a specialized language model in structured classification of findings. This hybrid approach of machine translation and expert validation enabled the creation of large-scale datasets while maintaining high-quality standards. This study provides essential resources for advancing medical artificial intelligence research in Japanese health care settings, using datasets and models publicly available for research to facilitate further advancement in the field.

## Introduction

Recent advances in large language models (LLMs) have demonstrated remarkable capabilities across various domains [1], thereby increasing the focus on developing multilingual models to serve diverse linguistic communities [2]. This trend is exemplified by the release of specialized language models, such as Gemma-2-JPN, a Japanese-specific variant of Google’s open LLM Gemma [3,4]. However, the development of such specialized models critically depends on the availability of high-quality domain-specific datasets in the target language. This requirement becomes particularly crucial in specialized fields, such as medical imaging [5-7], where the interpretation of diagnostic findings demands both technical precision and linguistic accuracy.

Computed tomography (CT) is indispensable in modern medical diagnostics, facilitating disease staging, lesion evaluation, and early detection. Japan has the highest number of CT scanners per capita and an annual scan volume surpassing that of most developed nations, presenting a vast reservoir of medical imaging data [8,9]. The extensive use of CT technology has positioned Japan as a pivotal contributor to global medical imaging resources. However, despite the proliferation of multilingual models and growing emphasis on language-specific adaptations, there remains a notable absence of large-scale Japanese radiology report datasets [10], which is a critical gap hindering the development of Japanese-specific medical language models.

To address this challenge, we constructed “CT-RATE-JPN,” a Japanese version of the extensive “CT-RATE” dataset [11], which consists of CT scans and interpretation reports collected from 21,304 patients in Turkey. Although general academic knowledge benchmarks have been successfully adapted for Japanese, as evidenced by JMMLU (Japanese Massive Multitask Language Understanding) [12] and JMMMU (Japanese Massive Multi-discipline Multimodal Understanding) [13], which are Japanese versions of MMLU (Massive Multitask Language Understanding) [14,15] and MMMU (Massive Multi-discipline Multimodal Understanding) [16], respectively, and medical benchmarks, such as JMedBench [17], have emerged by combining translated English resources and Japanese medical datasets, a large-scale Japanese dataset specifically focusing on radiology reports, remains notably absent.

CT-RATE-JPN uses an innovative dataset construction approach by leveraging LLM-based machine translation to efficiently generate a large volume of training data. This addresses the fundamental challenge of the dataset scale in medical artificial intelligence (AI) development while maintaining quality through a strategic validation approach. A subset of the data undergoes careful revision by radiologists to create a rigorously verified validation dataset. This dual-track methodology, which combines machine-translated training data with specialist-validated evaluation data, establishes a robust pipeline for both training data acquisition and performance evaluation.

Both CT-RATE and CT-RATE-JPN retain licenses that allow free use for research purposes, thereby supporting broader research initiatives in medical imaging and language processing. To demonstrate the practical use of the CT-RATE-JPN dataset, we developed CT-BERT-JPN, a deep learning–based language model specifically designed for extracting structured labels from Japanese radiology reports. By converting unstructured Japanese medical text into standardized, language-agnostic structured labels, CT-BERT-JPN provides a scalable framework for integrating Japanese radiology data into the global medical AI development, thereby addressing a critical need in the rapidly evolving landscape of multilingual medical AI.

## Methods

### Dataset Overview

**Figure 1.**
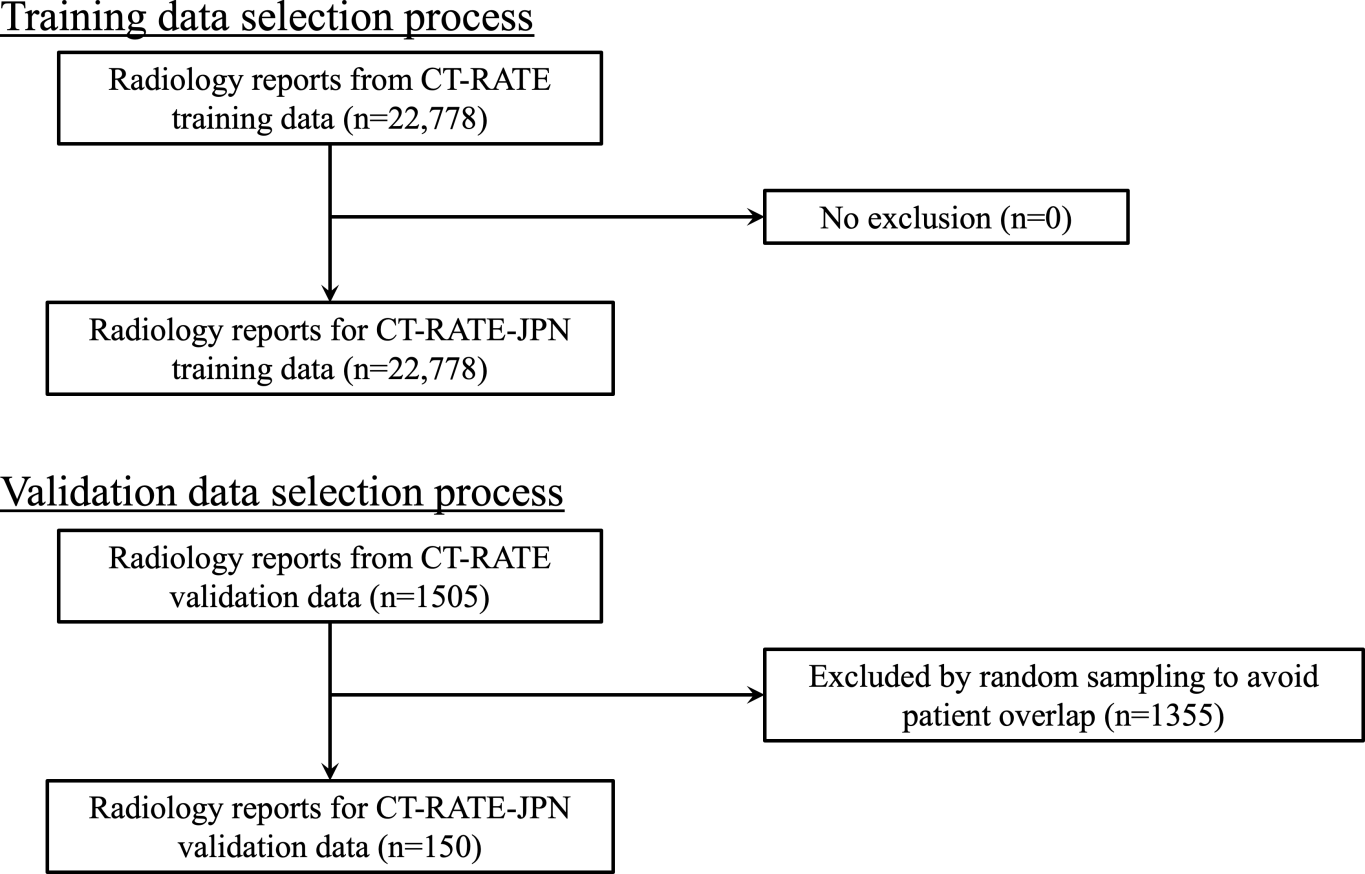
Data selection process for CT-RATE-JPN training and validation datasets. The training dataset included all 22,778 radiology reports from CT-RATE without exclusion. For the validation dataset, 150 reports were randomly sampled from 1505 available reports, with 1355 reports excluded to avoid patient overlap between training and validation sets.

CT-RATE is a comprehensive dataset comprising 25,692 noncontrast chest CT volumes of 21,304 unique patients from the Istanbul Medipol University Mega Hospital [11]. We selected this dataset because it is uniquely positioned as the only publicly available large-scale dataset that pairs CT volumes with radiology reports and permits the redistribution of derivative works. This dataset includes corresponding radiology text reports (consisting of a detailed findings section documenting observations and a concise impression section summarizing key information), multi-abnormality labels, and metadata. The dataset is divided into 2 cohorts, 20,000 patients for the training set and 1304 patients for the validation dataset, allowing robust model training and evaluation across diverse patient cases [18,19]. The training dataset, comprising 22,778 unique reports, was used in constructing CT-RATE-JPN, a Japanese-translated version of the dataset created using machine translation. Independently, we randomly sampled 150 reports from the validation cohort (n=1304); this validation subset was never used during model training and was reserved solely for inference-time evaluation. Those 150 reports were first machine translated and then manually revised and refined by radiologists. The data selection process is illustrated in Figure 1.

The CT-RATE dataset was annotated using 18 structured labels covering key findings relevant to chest CT analysis. These labels included “Medical material,” “Arterial wall calcification,” “Cardiomegaly,” “Pericardial effusion,” “Coronary artery wall calcification,” “Hiatal hernia,” “Lymphadenopathy,” “Emphysema,” “Atelectasis,” “Lung nodule,” “Lung opacity,” “Pulmonary fibrotic sequela,” “Pleural effusion,” “Mosaic attenuation pattern,” “Peribronchial thickening,” “Consolidation,” “Bronchiectasis,” and “Interlobular septal thickening.” The creators of the CT-RATE dataset developed a structured findings model based on the RadBERT architecture [20,21], trained on a manually labeled subset to label the remaining cases. This model achieved an *F*_1_-score ranging from 0.95 to 1.00, demonstrating its efficacy in accurately structuring radiological findings from CT reports. This approach underscores the reliability of CT-RATE’s structured annotations for developing high-performance diagnostic models. These structured labels were also used in the development of a Japanese structured findings model for CT-RATE-JPN, enabling the accurate structuring of radiological findings in Japanese CT reports.

### Ethical Considerations

Given that CT-RATE is a publicly available dataset with deidentified patient information, and that our study focused on the translation and linguistic analysis of the existing dataset without accessing any additional patient data, institutional review board approval was not required for this research.

### Translation for CT-RATE-JPN

**Figure 2.**
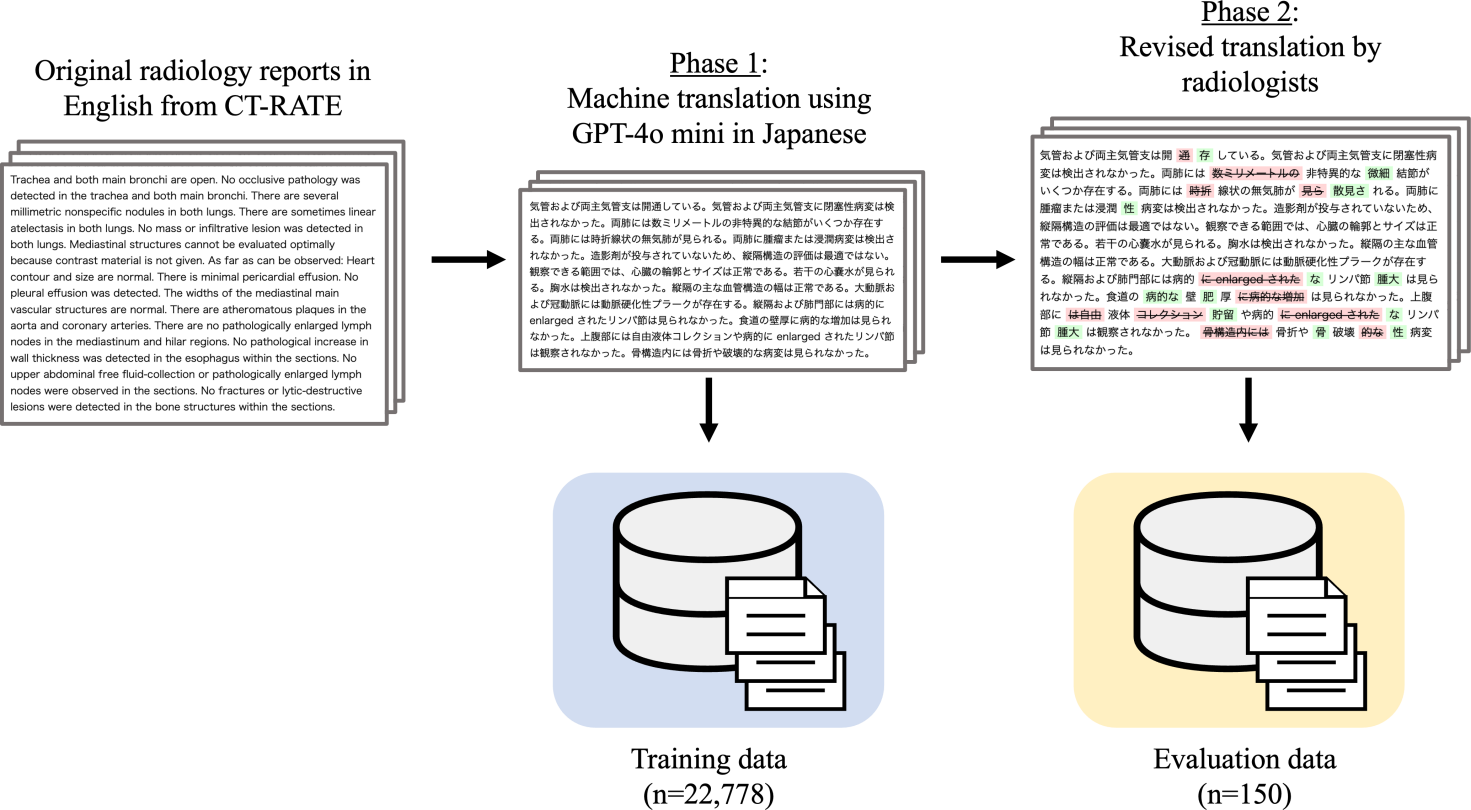
Workflow for translation and validation in constructing CT-RATE-JPN. The figure outlines machine translation application using GPT-4o mini for the training dataset and 2-phase manual correction for the 150 validation reports. Phase 1 involved initial revisions by radiology residents. Phase 2 consisted of expert review and refinement by board-certified radiologists.

For CT-RATE-JPN, machine translation was conducted using GPT-4o mini (API version, “gpt-4o-mini-2024-07-18”) [22], which is a lightweight, fast version of OpenAI’s GPT-4o model [23]. The GPT-4o mini produces high-accuracy translations at an affordable rate, making it suitable for large-scale dataset translations. Each radiological report was processed by translating the findings into short impression sections. The complete translation prompts used for GPT-4o mini are provided in Figures S1 (original Japanese prompt) and S2 (English translation) in Multimedia Appendix 1. Figure 2 shows the study workflow.

To ensure the accuracy and reliability of the evaluation data, we conducted a comprehensive manual correction process on 150 reports from the validation dataset. This process consisted of 2 distinct phases. In the first phase, we assembled a team of 5 radiology residents, all between their fourth and sixth postgraduate years, to conduct an initial review and revision of the machine translations. The reports were systematically distributed among team members to optimize workflow efficiency. We intentionally chose a larger team to incorporate diverse clinical perspectives and minimize potential translation bias during the review process. The second phase involved a thorough expert review by 2 board-certified radiologists with extensive experience (10 and 11 postgraduate years). The senior radiologists divided the revised translations into final confirmation and refinement. The structured approach to task allocation combined with this rigorous 2-step review process ensured that the validation dataset for CT-RATE-JPN met the high-quality standards necessary for a robust model assessment.

For the training dataset, all translations were generated using GPT-4o mini without manual correction. This dataset was specifically designed for machine learning model training. The decision to rely exclusively on machine-translated data for the training set balanced the scale and practical constraints of the manual annotation.

Both CT-RATE and CT-RATE-JPN were released under a Creative Commons Attribution (CC BY-NC-SA) license, allowing free use for noncommercial research purposes with proper citation and shared terms for derivative works.

### Development of CT-BERT-JPN for Structured Finding Classification

For model training, we randomly split the dataset in a 4:1 ratio, with 80% used for training and 20% for internal evaluation. The pretrained “tohoku-nlp/bert-base-japanese-v3” model from Hugging Face was used [24]. This model follows the architecture of the original BERT base model with 12 layers, 768 hidden dimensions, and 12 attention heads. It was pretrained on extensive Japanese datasets, including the Japanese portion of the CC-100 corpus [25,26] and Japanese version of Wikipedia [27]. BERT-based models have demonstrated significant success in downstream tasks in the medical domain [28,29], making them a promising choice for our research.

**Figure 3.**
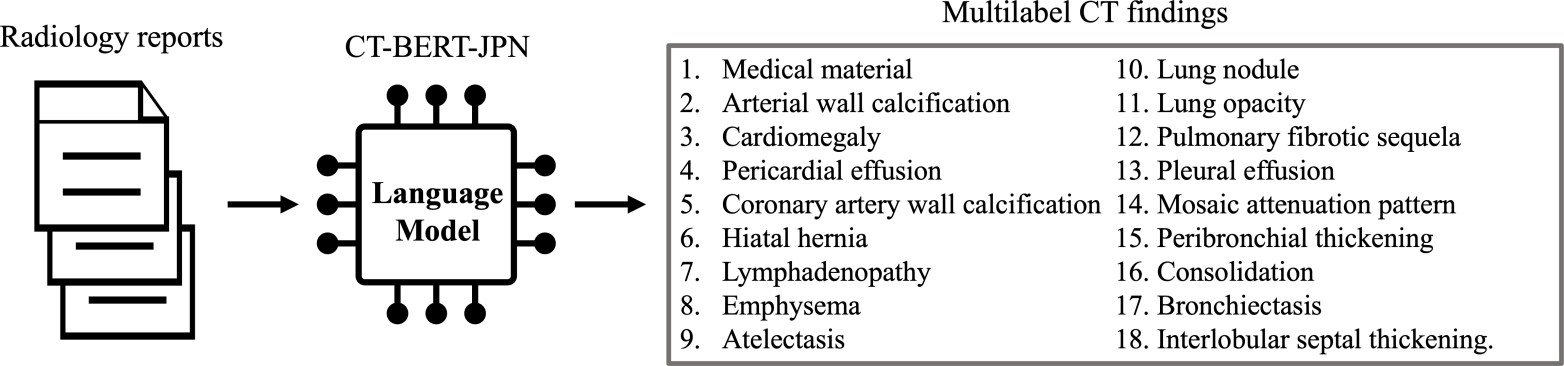
CT-BERT-JPN development workflow showing data preprocessing and model fine-tuning for structured computed tomography (CT) classification of findings.

Training was conducted on Google Colaboratory equipped with an NVIDIA L4 GPU using the transformers library (version 4.46.2) with a learning rate of 2×10⁻⁵, batch size of 8 for both training and evaluation, and weight decay of 0.01. Binary cross-entropy loss was applied to optimize the model for multilabel classification. Given the domain shift between the validation dataset (machine-translated) and test dataset (radiologist-revised), hyperparameter tuning was deliberately omitted to avoid overfitting, as such optimization may not contribute to performance improvement under domain shift conditions [30,31]. The model was trained over 4 epochs with internal evaluation and checkpoint savings at each epoch. The total training time was approximately 51.5 minutes (3090.54 s) across 4 epochs, with an average of 12.9 minutes (772.64 s) per epoch. The best-performing model on the internal evaluation data was selected and subsequently used for testing on the validation dataset of CT-RATE-JPN, to ensure a reliable performance assessment. Figure 3 shows the overall workflow for developing CT-BERT-JPN.

### Translated Radiology Reports Evaluation by Automatic Metrics

For basic text analysis, we examined the structural characteristics of the translated reports, including character, word, and sentence count, as well as lexical diversity. Unlike English, Japanese does not use spaces to delimit words. Therefore, we used MeCab (version 1.0.10) [32], one of the most widely used morphological analyzers for Japanese text processing, to accurately segment and count words. These metrics were calculated for both machine-translated and radiologist-revised texts to assess the consistency of textual characteristics across different stages of dataset creation.

For translation quality assessment, we computed the bilingual evaluation understudy (BLEU) [33] and recall-oriented understudy for gisting evaluation (ROUGE)-1, ROUGE-2, and ROUGE-L scores [34] using NLTK (version 3.9.1) and rouge-score (version 0.1.2) libraries.

The BLEU metric evaluates the accuracy of machine-translated text by comparing it to a reference translation. It measures how many words and short phrases in the machine translation match those in the reference translation, to assess the degree of similarity in wording and phrasing between the two texts.

The ROUGE metric assesses the quality of summaries or translations by measuring the overlap between machine-generated and reference texts. ROUGE-1 considers the overlap of individual words; ROUGE-2 examines the overlap of pairs of consecutive words; and ROUGE-L focuses on the longest matching sequence of words between two texts. These metrics emphasize recall by evaluating how much of the important content from the reference text is captured in the machine-generated text. These metrics were calculated by comparing machine-translated texts with radiologist-revised reference translations in the validation dataset.

### Translated Radiology Reports Evaluation by Radiologists

To assess the quality of machine-translated radiology reports, we implemented a systematic evaluation by radiologists. A subset of reports was randomly assigned to radiologists for review, with each report evaluated by 2 reviewers: 1 senior radiologist (designated as rater 1, PGY 6‐11) and 1 junior radiologist (designated as rater 2, PGY 4‐5). To maintain objectivity, radiologists were never assigned to evaluate reports they had previously reviewed or corrected.

The evaluation used a 5-point Likert scale (1=poor to 5=excellent) across 3 distinct dimensions:

1. Grammatical correctness: Assessment of linguistic accuracy, including proper Japanese grammar and natural language usage. Presence of nonexistent words, unnatural English terms, or grammatical errors resulted in lower scores. Specialized terminology errors were evaluated separately.
2. Medical terminology: Evaluation of the accuracy and appropriateness of medical and radiological terminology in Japanese. This dimension specifically focused on technical terms and domain-specific language.
3. Overall readability: Subjective assessment of how comprehensible and fluent the translated text appeared to radiologists in clinical practice. While this dimension relates to the previous two categories, it captured the holistic impression of the text’s utility in a clinical setting.

Inter-rater reliability was calculated using quadratic weighted kappa (QWK) to ensure consistency in evaluations across different radiologists, as this metric is particularly suitable for ordinal data such as Likert scales. This multi-dimensional evaluation approach provided comprehensive insights into both the technical accuracy and practical usability of machine-translated radiology reports in a Japanese clinical context.

### CT-BERT-JPN Performance Evaluation

To evaluate the performance of the classification model in CT findings extraction, we used a test dataset comprising 150 radiology reports revised by radiologists to ensure accuracy. The key metrics calculated for model assessment included accuracy, precision, recall, *F*_1_-score, area under the receiver operating characteristic curve (AUC-ROC), and average precision (AP). 95% CIs for these metrics were calculated using 1000 bootstrap resampling iterations. The Scikit-learn library (version 1.5.2) was used for the analyses.

### Baseline Comparison With GPT-4o

To establish a baseline, we performed structured labeling using GPT-4o (API version, “gpt-4o-2024-11-20”), as it is widely adopted in various radiology tasks as a representative closed-source commercial LLM. We specifically chose GPT-4o because, to our knowledge, no existing open-source models were available for this particular task at the time of the study. We used a zero-shot prompting strategy that instructed GPT-4o to extract binary labels (0/1) for each of the 18 target findings from Japanese radiology reports and output results in JSON format, without additional prompt engineering or few-shot examples. The input prompts used for GPT-4o are presented in Figures S3 (original Japanese version) and S4 (English translations) in Multimedia Appendix 1.

### Statistical Analysis

The statistical comparisons were analyzed using the Wilcoxon signed-rank test. This nonparametric test was applied in two contexts: (1) comparing paired predictions from CT-BERT-JPN and GPT-4o on identical validation cases, and (2) comparing CT-BERT-JPN performance between radiologist-revised translated reports versus raw machine-translated reports as input, where each pair consisted of model outputs on the same test sample under different conditions. The Wilcoxon signed-rank test was chosen as it does not require the assumption of normal distribution and is appropriate for comparing paired model outputs. Statistical analysis was conducted using the scipy.stats library (version 1.11.3).

## Results

### Dataset Overview

The basic text statistics of the translated reports are summarized in Table 1, in separate sections for Findings and Impression. The training (n=22,778) and validation datasets (consisting of 150 machine-translated reports and 150 radiologist-revised reports) showed a consistent text structure across all metrics.

**Table 1.** Text statistics on CT-RATE-JPN across different datasets, including “Findings” and “Impression.”

Section		n	Characters, mean (SD)	Words, mean (SD)	Sentences, mean (SD)	Unique words, mean (SD)
Findings						
Training (MT^a^)		22,778	467.0 (148.0)	303.3 (95.4)	15.5 (4.6)	126.8 (29.5)
Validation (MT^a^)		150	475.0 (130.1)	307.0 (83.6)	15.7 (3.9)	128.9 (27.8)
Validation (Refined^b^)		150	455.6 (122.6)	297.7 (80.8)	15.7 (4.0)	126.2 (27.4)
Impression						
Training (MT^a^)		22,778	89.1 (68.9)	55.7 (43.6)	3.1 (2.2)	38.1 (23.4)
Validation (MT^a^)		150	101.3 (76.0)	63.2 (48.1)	3.5 (2.6)	41.7 (24.3)
Validation (Refined^b^)		150	97.8 (72.8)	61.2 (46.0)	3.6 (2.6)	40.9 (24.0)

a MT: machine-translated text using GPT-4o mini.

b Refined indicates text subjected to radiologist review and refinement.

The Findings section had character counts averaging around 455.6‐475.0 characters, with slightly lower counts in radiologist-revised texts compared to machine translations. Word count followed a similar pattern, averaging approximately 300 words per report across all datasets. The Impression section was notably more concise, as expected from summary statements. Character counts averaged around 89.1‐101.3 characters, with word counts of approximately 55.7‐63.2 words per report. The sentence structure was also more condensed, with approximately 3.1‐3.6 sentences per report.

Notably, in both sections, the overall text structure remained consistent between machine-translated and radiologist-revised versions, with similar patterns in sentence length and organization. Although the refined versions had slightly lower character and word counts than their machine-translated counterparts, the basic structural characteristics of the reports were preserved throughout the translation and refinement processes.

**Figure 4.**
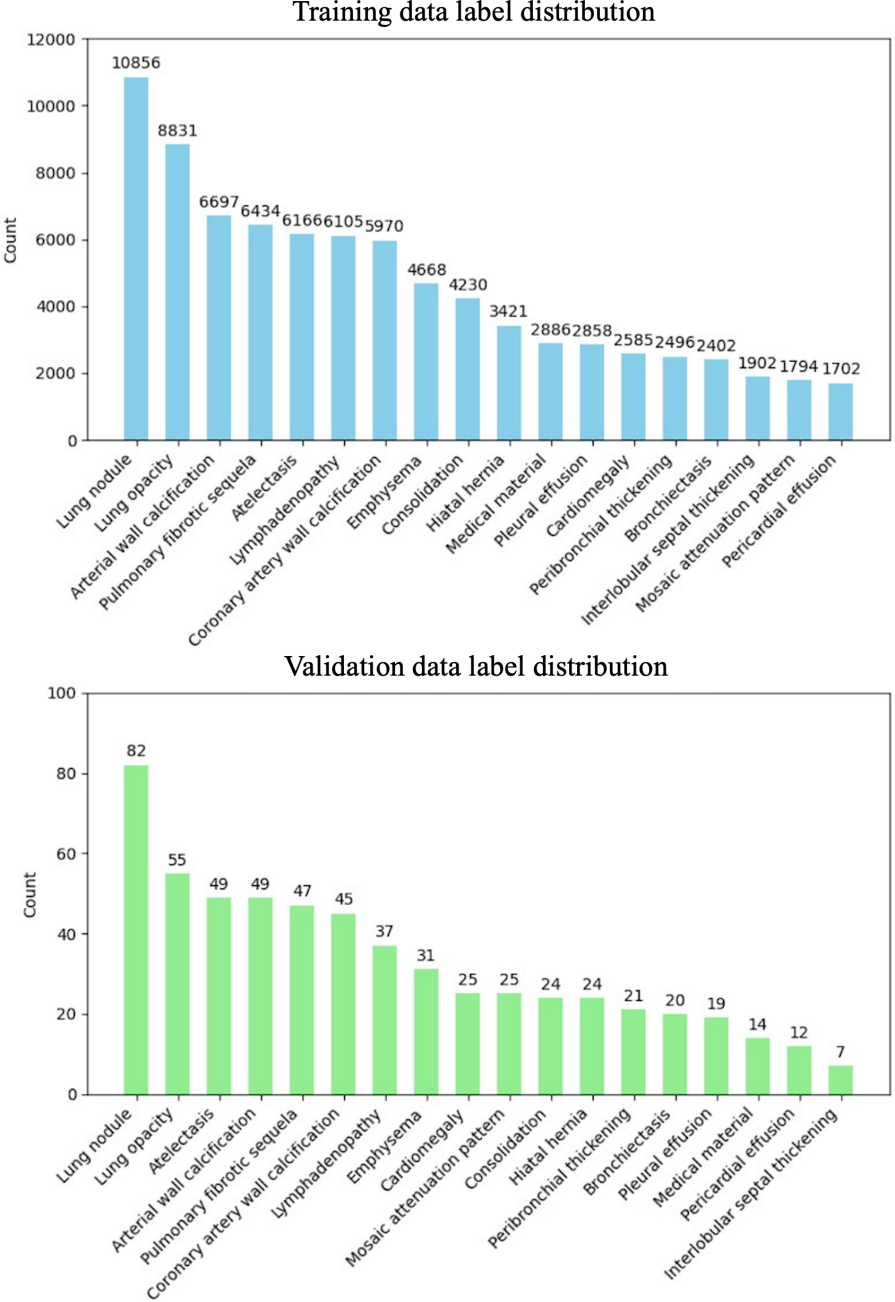
Bar plots showing the data distribution across different findings, sorted in descending order. Top: Training dataset distribution, Bottom: Validation dataset distribution. The number above each bar represents the number of positive samples for each condition.

Figure 4 shows that the analysis of the label distributions revealed a significant class imbalance in both the training and validation datasets. In the training set, “Lung nodule” appears most frequently with 10,856 instances, whereas “Interlobular septal thickening” occurs least frequently with only 1702 instances, representing a ratio of approximately 6.4:1. This imbalance is even more pronounced in the validation dataset, where the ratio between the most frequent (82) and least frequent (7) class instances is approximately 11.7:1.

### Translated Radiology Reports Evaluation

We evaluated the quality of machine-translated reports in CT-RATE-JPN using both automated metrics and expert assessments. For automated evaluation, we compared GPT-4o mini translations with radiologist-revised references in the validation dataset. The evaluation metrics for both sections are summarized in Table 2. These scores are high, indicating that the machine translation maintained the fundamental structure and meaning of the original reports.

**Table 2.** Automated evaluation metrics comparing GPT-4o mini translations with radiologist-revised references in the validation dataset.

Section	BLEU^a^, mean (SD)	ROUGE-1^b^, mean (SD)	ROUGE-2, mean (SD)	ROUGE-l, mean (SD)
Findings	0.731 (0.104)	0.876 (0.050)	0.770 (0.091)	0.854 (0.064)
Impression	0.690 (0.196)	0.857 (0.104)	0.748 (0.161)	0.837 (0.120)

a BLEU: bilingual evaluation understudy.

b ROUGE: recall-oriented understudy for gisting evaluation.

**Figure 5.**
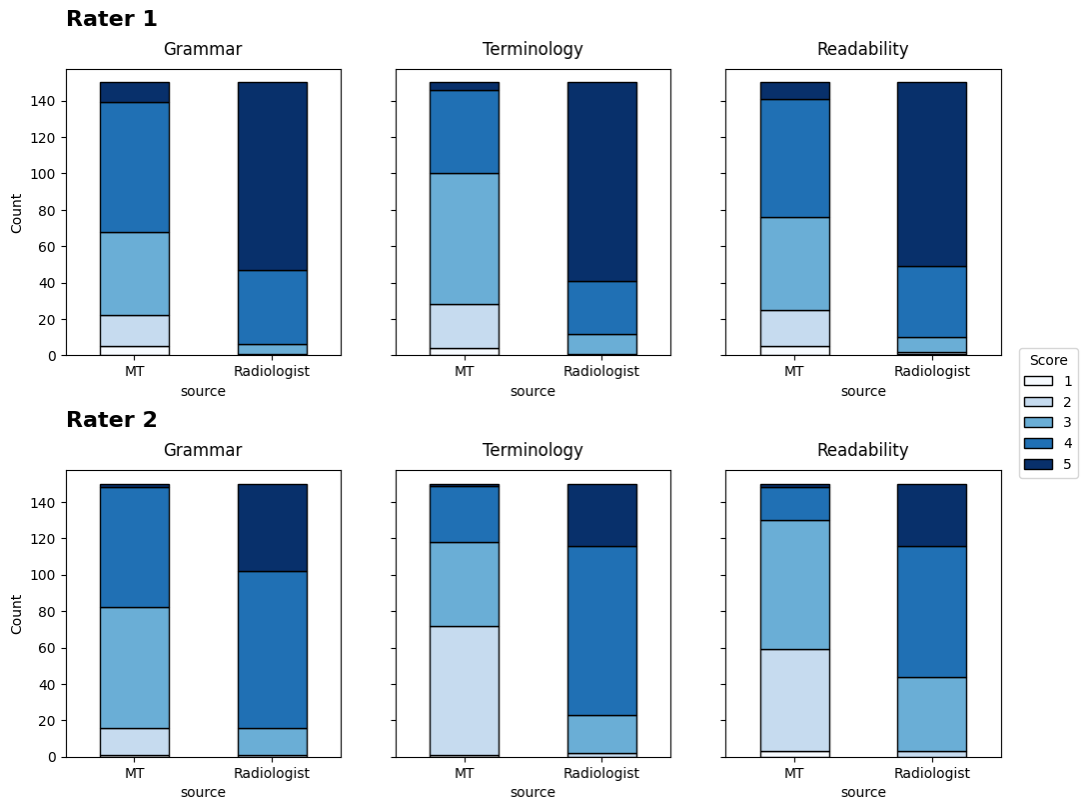
Distribution of evaluation scores for machine-translated (MT) and radiologist-revised translations across 3 dimensions (grammar, terminology, and readability) by 2 rater groups. The upper panel shows evaluations by senior radiologists (Rater 1, postgraduate year 6‐11) and the lower panel shows evaluations by junior radiologists (Rater 2, postgraduate year 4‐5). Scores were assessed using a 5-point Likert scale (1=poor to 5=excellent).

The evaluation of machine-translated radiology reports showed moderate inter-rater agreement with QWK values of 0.410 for grammar, 0.522 for terminology, and 0.408 for readability. These moderate agreement levels may reflect the inherent subjectivity of Likert-scale-based quality assessments. However, radiologist refinements resulted in substantial score improvements across all evaluation dimensions. The highest agreement between senior radiologists (Rater 1) and junior radiologists (Rater 2) was observed in terminology assessment. Figure 5 shows that both raters consistently scored radiologist-revised translations significantly higher than machine translations across all dimensions. Rater 1’s evaluations were consistently higher than Rater 2’s for both MT and radiologist-revised translations. Table 3 demonstrates that all improvements between MT and radiologist revisions were statistically significant (*P*<.001) across all evaluation criteria. While GPT-4o mini translations achieved moderate scores, radiologist revisions provided significant enhancements, particularly in terminology accuracy. This highlights the value of domain expertise in medical translation, even when using advanced language models. In addition, Table 4 shows the distribution of quality changes, with the majority of radiologist revisions (65%‐91% depending on rater and dimension) resulting in improved ratings compared to machine translation, while deterioration was minimal (0%‐6% across all categories).

**Table 3.** Evaluation of translated texts comparing mean Likert scale scores of GPT-4o mini translations (MT) and radiologist-revised translations.

Evaluation criteria		MT^a^, mean (SD)	Radiologist, mean (SD)	*P* value^b^
Rater 1				
Grammar		3.44 (0.91)	4.64 (0.58)	<.001
Terminology		3.15 (0.81)	4.64 (0.65)	<.001
Readability		3.35 (0.91)	4.59 (0.69)	<.001
Rater 2				
Grammar		3.35 (0.71)	4.21 (0.64)	<.001
Terminology		2.73 (0.82)	4.06 (0.65)	<.001
Readability		2.73 (0.75)	3.91 (0.76)	<.001

a MT: machine-translated text using GPT-4 mini.

b *P* values were calculated using the Wilcoxon signed-rank test.

**Table 4.** Distribution of quality changes between machine translation and radiologist-revised translations across grammar, terminology, and readability dimensions.

Item	Rater 1			Rater 2		
	Worse	No change	Better	Worse	No change	Better
Grammar, n (%)	4 (2.7)	28 (18.7)	118 (78.7)	9 (6)	43 (28.7)	98 (65.3)
Terminology, n (%)	0 (0)	14 (9.3)	136 (90.7)	7 (4.7)	34 (22.7)	109 (72.7)
Readability, n (%)	1 (0.7)	29 (19.3)	120 (80)	9 (6)	30 (20)	111 (74)

**Figure 6.**
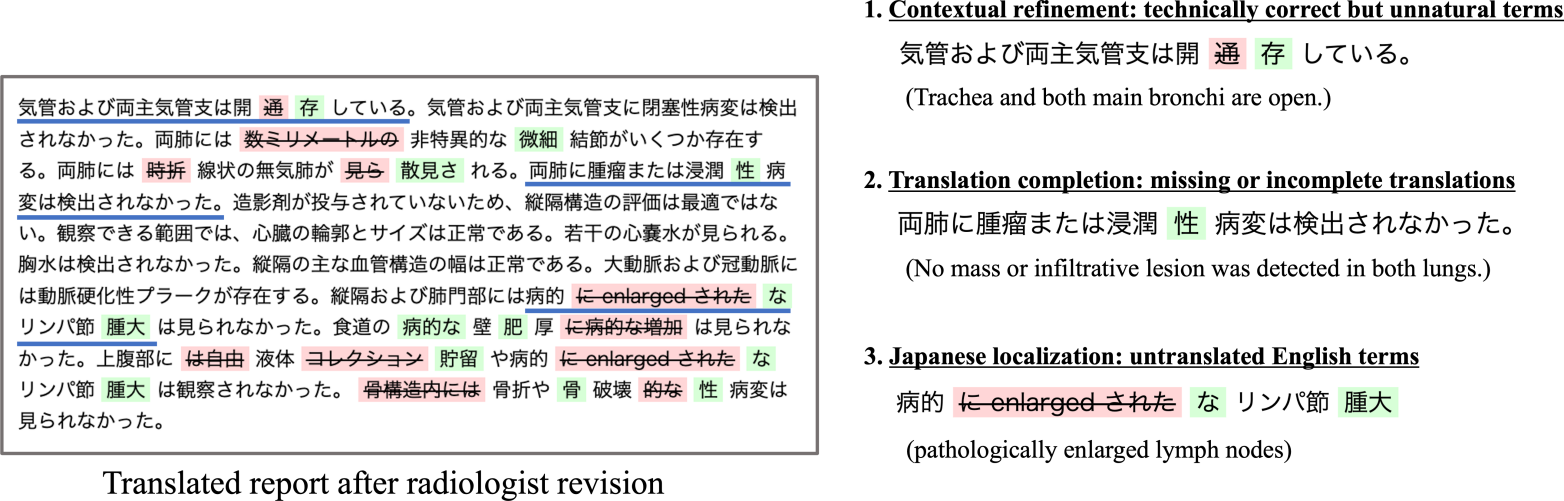
Representative examples of (computed tomography) CT report translation improvements in 3 categories: contextual term refinement, completion of missing details, and localization of untranslated terms from English to Japanese radiological equivalents.

Despite the high automated metric scores (see Table 2), expert scoring evaluation by radiologists revealed that substantial revisions were necessary for medical terminology, with significant improvements observed between machine translations and radiologist-revised versions. Radiologists’ assessments identified the representative patterns of necessary modifications. This expert review revealed 3 major categories of improvements (see Figure 6; the original English reports used are shown in Figure S5 in Multimedia Appendix 1), which reflect typical challenges in medical translation: (1) contextual refinement of technically correct but unnatural terms, (2) completion of missing or incomplete translations, and (3) Japanese localization of untranslated English terms. Although not exhaustive, these patterns represent key areas where human expertise complements machine translation in medical contexts.

The first category, contextual refinement, involved replacing technically accurate but clinically uncommon expressions with more natural medical terminology. For instance, direct translations of anatomical conditions were often revised to their proper radiological equivalents when describing vessel status, reflecting standard terminology in Japanese clinical practice. The second category addresses cases in which certain medical terms were either missing or incompletely translated, thereby requiring additional context-specific information. A typical example would be anatomical descriptions lacking specific diagnostic terminology that is common in radiological reporting. The third category focuses on proper localization of English medical terms that were initially left untranslated, such as converting technical descriptors of pathological findings into appropriate Japanese radiological counterparts.

### CT-BERT-JPN Performance Evaluation

The CT-BERT-JPN model achieved a micro *F*_1_-score of 0.9607 on the internal evaluation data, which served as the model selection criterion. When evaluated on the validation dataset, the model showed a micro *F*_1_-score of 0.9695 with machine-translated inputs and 0.9520 with radiologist-revised inputs, representing a performance decrease of 0.0175. Table 5 presents the performance evaluation results of CT-BERT-JPN across 18 different findings from 150 chest CT radiological reports on the validation dataset. The model achieved perfect scores (1.000) across all evaluation metrics (accuracy, precision, recall, *F*_1_-score, AUC-ROC, and AP) for pericardial effusion, hiatal hernia, and mosaic attenuation patterns. It demonstrated high accuracy exceeding 0.950 in 17 out of 18 findings, with AUC-ROC values surpassing 0.98 in all findings. Furthermore, AP remained consistently strong—ranging from 0.873 to 1.000—despite the imbalanced distribution of positive samples (7 cases for interlobular septal thickening up to 82 cases for lung nodules), underscoring the model’s robust discriminative ability across all conditions.

**Table 5.** Performance evaluation of CT-BERT-JPN across 18 different findings.

Findings	Accuracy (95% CI)	Precision (95% CI)	Recall (95% CI)	*F*_1_-score (95% CI)	AUC-ROC^a^ (95% CI)	AP^b^
Medical material	0.973 (0.940‐0.993)	0.778 (0.538‐0.952)	1.000 (1.000‐1.000)	0.875 (0.700‐0.976)	0.999 (0.995‐1.000)	0.990 (0.950‐1.000)
Arterial wall calcification	0.987 (0.967‐1.000)	0.961 (0.898‐1.000)	1.000 (1.000‐1.000)	0.980 (0.946‐1.000)	1.000 (1.000‐1.000)	1.000 (1.000‐1.000)
Cardiomegaly	0.987 (0.967‐1.000)	1.000 (1.000‐1.000)	0.920 (0.800‐1.000)	0.958 (0.889‐1.000)	0.999 (0.996‐1.000)	0.996 (0.981‐1.000)
Pericardial effusion	1.000 (1.000‐1.000)	1.000 (1.000‐1.000)	1.000 (1.000‐1.000)	1.000 (1.000‐1.000)	1.000 (1.000‐1.000)	1.000 (1.000‐1.000)
Coronary artery wall calcification	0.987 (0.967‐1.000)	0.978 (0.927‐1.000)	0.978 (0.925‐1.000)	0.978 (0.943‐1.000)	1.000 (0.999‐1.000)	1.000 (0.997‐1.000)
Hiatal hernia	1.000 (1.000‐1.000)	1.000 (1.000‐1.000)	1.000 (1.000‐1.000)	1.000 (1.000‐1.000)	1.000 (1.000‐1.000)	1.000 (1.000‐1.000)
Lymphadenopathy	0.987 (0.967‐1.000)	0.973 (0.907‐1.000)	0.973 (0.912‐1.000)	0.973 (0.929‐1.000)	0.994 (0.983‐1.000)	0.987 (0.963‐1.000)
Emphysema	0.980 (0.953‐1.000)	0.938 (0.844‐1.000)	0.968 (0.889‐1.000)	0.952 (0.881‐1.000)	0.989 (0.970‐1.000)	0.960 (0.888‐1.000)
Atelectasis	0.993 (0.973‐1.000)	0.980 (0.929‐1.000)	1.000 (1.000‐1.000)	0.990 (0.963‐1.000)	1.000 (1.000‐1.000)	1.000 (1.000‐1.000)
Lung nodule	0.967 (0.940‐0.993)	0.975 (0.937‐1.000)	0.963 (0.921‐1.000)	0.969 (0.942‐0.994)	0.991 (0.978‐1.000)	0.994 (0.985‐1.000)
Lung opacity	0.953 (0.913‐0.987)	0.929 (0.857‐0.983)	0.945 (0.880‐1.000)	0.937 (0.885‐0.977)	0.991 (0.980‐0.999)	0.985 (0.963‐0.999)
Pulmonary fibrotic sequela	0.953 (0.920‐0.987)	0.935 (0.860‐1.000)	0.915 (0.833‐0.980)	0.925 (0.867‐0.974)	0.981 (0.960‐0.997)	0.973 (0.942‐0.995)
Pleural effusion	0.987 (0.967‐1.000)	0.905 (0.762‐1.000)	1.000 (1.000‐1.000)	0.950 (0.865‐1.000)	1.000 (0.997‐1.000)	0.997 (0.983‐1.000)
Mosaic attenuation pattern	1.000 (1.000‐1.000)	1.000 (1.000‐1.000)	1.000 (1.000‐1.000)	1.000 (1.000‐1.000)	1.000 (1.000‐1.000)	1.000 (1.000‐1.000)
Peribronchial thickening	0.960 (0.927‐0.987)	1.000 (1.000‐1.000)	0.714 (0.529‐0.905)	0.833 (0.692‐0.950)	0.985 (0.960‐1.000)	0.948 (0.869‐0.997)
Consolidation	0.933 (0.893‐0.973)	0.706 (0.533‐0.862)	1.000 (1.000‐1.000)	0.828 (0.696‐0.926)	0.996 (0.988‐1.000)	0.985 (0.951‐1.000)
Bronchiectasis	0.980 (0.953‐1.000)	0.870 (0.714‐1.000)	1.000 (1.000‐1.000)	0.930 (0.833‐1.000)	0.990 (0.970‐1.000)	0.873 (0.686‐1.000)
Interlobular septal thickening	0.993 (0.980‐1.000)	0.875 (0.600‐1.000)	1.000 (1.000‐1.000)	0.933 (0.750‐1.000)	1.000 (1.000‐1.000)	1.000 (1.000‐1.000)

a AUC-ROC: area under the receiver operating characteristic curve.

b AP: average precision.

compares the *F*_1_-scores of CT-BERT-JPN and GPT-4o across the 18 conditions, displayed as bar charts. Table S1 in Multimedia Appendix 1 presents the detailed results for GPT-4o across the 18 conditions using valid data. CT-BERT-JPN had higher *F*_1_-scores than GPT-4o in 11 findings and achieved equivalent performance in 2 findings, specifically the hiatal hernia and mosaic attenuation patterns, both of which attained perfect *F*_1_-scores. Notably, CT-BERT-JPN showed a higher performance in lymphadenopathy (0.142 higher) and atelectasis (0.074 higher), with statistically significant differences confirmed by the Wilcoxon signed-rank test (*P*=.003 and *P*=.005, respectively). However, the model performed poorly in 5 findings—most notably peribronchial thickening (–0.051) and consolidation (–0.035)—although Wilcoxon signed-rank tests did not detect statistically significant differences. Detailed performance metrics for GPT-4o are presented in Table S1 in Multimedia Appendix 1.

A detailed analysis of the performance of CT-BERT-JPN was conducted by comparing 2 scenarios: radiologist-revised translated reports versus raw machine-translated reports as input. The differences in the metrics are detailed in Table 6 (the performance metrics for machine translation can be found in Table S2 in Multimedia Appendix 1). The analysis showed that for most findings, performance differences were less than 5% with no significant variations, confirming the robustness of the model. However, peribronchial thickening showed notable decreases, with a decrease in recall of 0.238 and *F*_1_-score reduction of 0.143. Statistical analysis using the Wilcoxon signed-rank test revealed a significant difference (*P*=.025). Similarly, consolidation experienced relatively significant performance declines, with precision decreasing by 0.179 and *F*_1_-score by 0.092, with the Wilcoxon signed-rank test showing a significant difference (*P*=.034).

**Table 6.** Performance differences between CT-BERT-JPN models trained on radiologist-revised versus machine-translated inputs across multiple metrics.

Findings	Accuracy	Precision	Recall	*F*_1_-score	AUC-ROC^a^	AP^b^	*P* value^c^
Medical material	0.000^d^	−0.034	0.071	0.008	0.002	0.013	1.00
Arterial wall calcification	−0.006	−0.019	0.000	−0.010	0.000	0.000	.32
Cardiomegaly	−0.013	0.000	−0.080	−0.042	−0.001	−0.004	.16
Pericardial effusion	0.007	0.000	0.083	0.043	0.000	0.000	.32
Coronary artery wall calcification	0.000	0.000	0.000	0.000	0.000	0.000	N/A^e^
Hiatal hernia	0.000	0.000	0.000	0.000	0.000	0.000	N/A^e^
Lymphadenopathy	−0.006	−0.001	−0.027	−0.014	−0.006	−0.012	.32
Emphysema	−0.007	−0.030	0.000	−0.016	−0.011	−0.039	.56
Atelectasis	−0.007	−0.020	0.000	−0.010	0.000	0.000	.32
Lung nodule	−0.013	−0.025	0.000	−0.012	0.000	0.000	.16
Lung opacity	−0.007	−0.016	0.000	−0.008	−0.002	-0.004	.71
Pulmonary fibrotic sequela	−0.007	0.017	-0.042	−0.013	−0.005	-0.007	.71
Pleural effusion	−0.006	−0.045	0.000	−0.024	0.002	0.008	.32
Mosaic attenuation pattern	0.000	0.000	0.000	0.000	0.000	0.000	N/A^e^
Peribronchial thickening	−0.033	0.000	−0.238	−0.143	−0.010	-0.033	.03
Consolidation	−0.040	−0.179	0.042	−0.092	0.004	0.020	.03
Bronchiectasis	0.007	0.037	0.000	0.021	-0.006	-0.099	.32
Interlobular septal thickening	−0.007	−0.125	0.000	−0.067	0.000	0.000	.32

a AUC-ROC: area under the receiver operating characteristic curve.

b AP: average precision.

c *P* values were calculated using the Wilcoxon signed-rank test.

d Positive values indicate higher performance with radiologist-corrected translations, whereas negative values “-” indicate higher performance with raw machine translations.

e N/A indicates that the test was not performed because the predicted values were identical.

Our qualitative analysis of CT-BERT-JPN’s performance revealed several illustrative examples of how translation quality impacts model predictions. For peribronchial thickening, we observed cases where CT-BERT-JPN incorrectly predicted negative results for truly positive cases when using radiologist-revised translations. A notable example involved subtle terminological changes from “bilateral peribronchial thickening is observed” in the machine translation to “bilateral bronchial wall thickening is observed” in the radiologist-revised version, where this minor linguistic variation caused the model to misclassify a positive case as negative, demonstrating how nuanced expression differences can significantly impact the model’s detection performance. Conversely, for pericardial effusion, we found instances where radiologist refinement improved model accuracy, such as cases where “pericardial effusion” was incorrectly translated as “pleural effusion” in machine-translated reports, while radiologist refinement correctly rendered it as “fluid accumulation in the pericardial recess,” leading to accurate CT-BERT-JPN predictions that would have been incorrect with the raw machine translation. In addition, for medical material—the condition with the third-lowest *F*_1_-score—we identified systematic prediction errors that persisted regardless of translation quality, particularly in cases containing expressions such as “changes associated with tracheostomy are observed,” where both machine-translated and radiologist-revised versions retained identical phrasing, yet CT-BERT-JPN consistently predicted positive when the correct label should have been negative. While the presence of an endotracheal tube following tracheostomy would warrant a positive prediction for medical material, the description alone does not explicitly indicate the current presence of medical devices, suggesting that the model may be confounded by artificial interventions mentioned in the text, leading to false positive predictions based on procedural context rather than actual device presence.

## Discussion

### Principal Findings

The 3 key findings of our study regarding the development of Japanese medical imaging resources and analytical capabilities are:

First, an efficient workflow that combines machine translation with expert validation was established to successfully create a large-scale Japanese radiology dataset, maintaining high-quality standards through a radiologist’s focused review.

Second, despite its relatively compact architecture, our specialized CT-BERT-JPN model demonstrated superior performance to GPT-4o in most structured finding extraction tasks, highlighting the effectiveness of domain-specific optimization.

Third, the model maintained robust performance across both machine-translated and radiologist-revised reports, suggesting the viability of machine translation for training data creation in specialized medical domains.

### Dataset Development and Quality Assessment

In the field of medical imaging datasets, several English-language resources have been previously established, including the OpenI dataset [35] and MIMIC-CXR [36] for chest radiographs, and AMOS-MM [37] for chest-to-pelvic CT scans; however, these datasets are exclusively available in English, with limited multilingual adaptations. Although many LLMs and VLMs have multilingual capabilities [26,38,39], their performance is consistently degraded when handling non-English languages, including Japanese [40]. A significant factor contributing to this performance gap is the scarcity of non-English datasets, making the development of such resources an urgent priority.

Machine-translated reports showed minimal differences in basic structural elements compared with the original texts, with word counts and sentence lengths varying by less than 5%. Evaluation of translation quality using the BLEU and ROUGE metrics also demonstrated high performance, indicating that the overall structure and content of the reports were well preserved. However, a Likert-scale evaluation by radiologists on grammar, medical terminology, and readability revealed that raw GPT-4o mini outputs still required substantial refinement in all 3 areas. Radiologist-led corrections efficiently addressed these shortcomings, significantly boosting translation quality for our validation dataset. This limitation necessitated a rigorous refinement process involving expert radiologists, which proved crucial for establishing a reliable validation dataset.

Our efficient workflow enabled the review and correction of approximately 70,000 characters of machine-translated text, significantly reducing the time and effort required for extensive translation verification. This efficiency was achieved through a structured review process in which radiologists would focus primarily on medical terminology and critical semantic elements rather than reviewing every aspect of the translation. This systematic approach, which combines large language models with domain expert validation, presents a scalable methodology for dataset creation between distinctly different languages, such as English and Japanese. This hybrid process offers a promising framework that can be adapted not only to other languages but also to specialized domains beyond radiology, where precise terminology and domain expertise are critical.

### Model Performance and Evaluation

When combined with CT volumes, CT-BERT-JPN demonstrated significant potential for various text-based applications in medical imaging analysis. Our comprehensive evaluation revealed exceptional performance in structured extraction of findings across diverse radiological conditions, with *F*_1_-score consistently above 0.95 and perfect scores in several findings. More notably, CT-BERT-JPN outperformed GPT-4o in 11 out of 18 findings (61%), achieving higher *F*_1_-score in significant conditions such as lymphadenopathy (+0.142) and atelectasis (+0.074). This superior performance is particularly remarkable considering that CT-BERT-JPN has approximately 110 million parameters, whereas recent large language models often employ hundreds of billions of parameters [41]. Our results demonstrate that a specialized, compact language model can achieve state-of-the-art performance in domain-specific tasks, even when compared with more sophisticated general-purpose models. While promising, our results should be interpreted cautiously given the limited validation dataset of 150 reports and notable class imbalance for certain findings (eg, 7 cases for interlobular septal thickening, 12 cases for pericardial effusion). Although we used both *F*_1_-scores and AP metrics to address data imbalance, the 95% CIs reveal substantial variability for rare findings (eg, interlobular septal thickening: 0.933 [0.750‐1.000]), where even high *F*_1_-scores may be statistically unstable due to small sample sizes. Larger-scale validation studies will be necessary for more definitive performance assessment.

Furthermore, the model maintained stable performance across both unmodified machine translations and radiologist-revised reports. While some conditions showed moderate performance variations between machine-translated and radiologist-revised reports, particularly in findings where machine translation errors were common (*F*_1_-score differences of -0.143 for peribronchial thickening and −0.092 for consolidation), with consolidation being notably affected by mistranslations of the related “infiltration,” the overall robustness of the model suggests that effective clinical applications can be developed using machine-translated training data.

To contextualize our results within the broader radiology NLP field, we note that established models, such as RadGraph [42] and the CheXpert labeling tool [43], have demonstrated strong performance with English reports. Particularly relevant is the CheXpert labeler, which tackles the most similar task of multilabel finding classification in radiology reports, achieving *F*_1_-scores ranging from 0.647 to 0.996 across different findings in chest X-ray analysis. Our CT-BERT-JPN shows comparable performance levels for the distinct task of Japanese CT finding extraction. While acknowledging the differences in imaging modality, language, and validation dataset size, these results represent promising performance and constitute the first openly available Japanese model for multilabel CT findings extraction. Nevertheless, because CT-BERT-JPN was trained and evaluated on a single, translation-derived dataset, its performance could degrade under real-world Japanese radiology reports due to domain shift.

### Clinical Implementation and Impact

Previous studies have explored hybrid approaches that combine human and machine translations in the medical domain. Early research demonstrated the effectiveness of combining preexisting translation databases with machine translation for medical guidelines, particularly in updating existing documents [44]. Another study reported challenges in the quality of machine translation from English to Chinese of public health materials caused by linguistic structural differences [45]. However, recent advances in LLMs have been remarkable, with studies showing high performance in radiology report translation [46]. Our research successfully developed effective datasets through a combination of machine and expert translation. This approach enables efficient dataset construction even for languages with limited medical data, representing a significant advancement in multilingual medical data development.

The development of such a structured findings extraction model in Japan, where medical imaging usage rates rank among the highest globally, is expected to contribute substantially to both domestic health care advancements and international model development. Our implementation incorporated previous studies on Japanese radiology report processing, including end-to-end approaches for clinical information extraction [47], natural language processing systems for pulmonary nodule follow-up assessment [48], and BERT-based transfer learning methods for anatomical classification [49].

Our multilabel classification approach addresses several implementation challenges unique to Japanese health care. These include vocabulary standardization across institutions, integration with existing reporting systems, and development of specialized Japanese medical vocabulary handling mechanisms. The ability of the model to process Japanese-specific medical expressions and maintain high performance across different reporting styles demonstrates its potential for broad clinical applications. However, prospective validation across multiple Japanese institutions remains essential for both performance evaluation and capturing institution-specific reporting patterns for targeted model improvements.

### Limitations and Future Directions

Several limitations exist in our study. First, although we demonstrated the effectiveness of our language model through rigorous evaluation, the usability of CT-RATE-JPN for vision-language models (such as CT-contrastive language image pre-training [CLIP] [18]), which require joint learning of CT volumes and text descriptions, remains to be empirically validated. Second, our reliance on the CT-RATE dataset may have introduced inherent biases in reporting styles and patterns, because radiology reports vary considerably across institutions and individual radiologists. Third, despite expert validation, the translation-based approach may not fully capture the nuanced expressions and specialized terminology commonly used in Japanese clinical practice. In addition, well-established parallel corpora for translation in the Japanese radiology domain are currently lacking, although projects such as “JMED-DICT” are working to address this gap by developing a comprehensive medical terminology dictionary of approximately 400,000 terms through the integration of existing medical dictionaries and resources [50]. Fourth, potential biases may stem from the multi-step translation process and dataset origin. The CT-RATE dataset originates from Turkish medical institutions and was translated into English before our Japanese translation. This process may introduce biases from demographic differences between Turkish and Japanese patient populations, Turkish-specific reporting styles that may not align with Japanese clinical practices, and linguistic biases accumulated through successive translations, despite our refinement procedures. These biases may contribute to inflated performance metrics for CT-BERT-JPN that do not accurately reflect the model’s expected performance on native Japanese radiology reports in real-world settings.

Building upon these considerations, several promising directions for future research emerge. The primary direction is the development of vision language models using CT-RATE-JPN in conjunction with the CT volumes of CT-RATE. Given the successful development of Japanese CLIP models for general domain tasks, which have demonstrated the feasibility of cross-lingual vision-language alignment in Japanese [51], extending this approach to medical imaging is particularly promising. While this endeavor requires substantial computational resources and sophisticated training strategies, various approaches can be explored, such as additional training on existing models, for example, CT-CLIP. Furthermore, the construction of a new dataset comprising pairs of Japanese radiology reports and CT volumes from Japanese medical institutions can enable a more direct assessment of model performance in the Japanese healthcare context and potentially reveal insights unique to this setting. Our benchmark dataset, validated by radiologists through a systematic review process, provides a valuable foundation for evaluating such Japanese-English and English-Japanese translation models in radiology.

### Conclusion

In this study, we introduced CT-RATE-JPN, a comprehensive Japanese dataset of CT interpretation reports, and developed a specialized language model for structured labeling. Our model demonstrated superior performance compared with GPT-4o, achieving higher *F*_1_-scores in numerous categories of structured findings extraction. The creation of CT-RATE-JPN, along with our publicly available structured findings model, represents a significant contribution to Japanese medical imaging research. By making both the dataset and model freely accessible to the research community, we enable reproducibility and foster collaborative advancement in the field. This work not only provides essential resources for the medical AI community but also establishes a robust foundation for developing more sophisticated multilingual medical vision-language models. These openly available contributions are expected to support the development of AI-assisted diagnostic tools, while maintaining the high standards required for clinical applications in radiology.

## Supplementary material

10.2196/71137 Multimedia Appendix 1 Supplementary materials including detailed performance evaluation tables for GPT-4o and CT-BERT-JPN across 18 pathological findings, machine translation and structured labeling prompts used in the study (both Japanese and English versions), and sample radiology reports.

## Abbreviations

AI: artificial intelligence
AP: average precision
AUC: area under the curve
BERT: Bidirectional Encoder Representation from Transformers
BLEU: bilingual evaluation understudy
BY-NC-SA: Creative Commons Attribution-NonCommercial-ShareAlike
CLIP: Contrastive Language Image Pre-training
CT: computed tomography
JMMLU: Japanese Massive Multitask Language Understanding
JMMMU: Japanese Massive Multi-discipline Multimodal Understanding
LLM: large language model
MMLU: Massive Multitask Language Understanding
MMLU: Massive Multitask Language Understanding
MMMU: Massive Multi-discipline Multimodal Understanding
MMMU: Massive Multi-discipline Multimodal Understanding
NLTK: Natural Language ToolKit
PGY: postgraduate year
QWK: quadratic weighted kappa
ROC: receiver operating characteristic
ROUGE: Recall-Oriented Understudy for Gisting Evaluation
VLM: Vision Language Model
