# Supplementary material for: Development of a Large-Scale Dataset of Chest Computed Tomography Reports in Japanese and a High-Performance Finding Classification Model: Dataset Development and Validation Study
Source: JMIR Med Inform. 2025 Aug 28;13:e71137. doi: 10.2196/71137 (PMC12392688; doi:10.2196/71137)

**Table S1.** Performance evaluation of GPT-4o across 18 different pathological findings and anatomical structures. The table shows the model's performance metrics, including Accuracy, Precision, Recall, and F1 score. *P* values were calculated using the Wilcoxon signed-rank test for comparison with CT-BERT-JPN. Values in parentheses represent 95% confidence intervals.

| Findings | Accuracy | Precision | Recall | F1 | *P* values |
| --- | --- | --- | --- | --- | --- |
| Medical material | 0.967 (0.933–0.993) | 0.737 (0.500–0.933) | 1.000 (1.000–1.000) | 0.848 (0.667–0.966) | .32 |
| Arterial wall calcification | 0.993 (0.980–1.000) | 0.980 (0.932–1.000) | 1.000 (1.000–1.000) | 0.990 (0.965–1.000) | .56 |
| Cardiomegaly | 0.993 (0.980–1.000) | 1.000 (1.000–1.000) | 0.960 (0.869–1.000) | 0.980 (0.930–1.000) | .56 |
| Pericardial effusion | 0.993 (0.980–1.000) | 1.000 (1.000–1.000) | 0.917 (0.714–1.000) | 0.957 (0.833–1.000) | .32 |
| Coronary artery wall calcification | 0.987 (0.967–1.000) | 1.000 (1.000–1.000) | 0.956 (0.887–1.000) | 0.977 (0.940–1.000) | 1.00 |
| Hiatal hernia | 1.000 (1.000–1.000) | 1.000 (1.000–1.000) | 1.000 (1.000–1.000) | 1.000 (1.000–1.000) | NA |
| Lymphadenopathy | 0.927 (0.880–0.967) | 0.964 (0.880–1.000) | 0.730 (0.583–0.867) | 0.831 (0.714–0.919) | <.01 |
| Emphysema | 0.980 (0.953–1.000) | 0.967 (0.889–1.000) | 0.935 (0.833–1.000) | 0.951 (0.880–1.000) | 1.00 |
| Atelectasis | 0.940 (0.900–0.973) | 0.845 (0.746–0.929) | 1.000 (1.000–1.000) | 0.916 (0.855–0.963) | <.01 |
| Lung nodule | 0.953 (0.920–0.980) | 0.963 (0.915–1.000) | 0.951 (0.903–0.988) | 0.957 (0.924–0.983) | .32 |
| Lung opacity | 0.933 (0.887–0.973) | 0.895 (0.811–0.967) | 0.927 (0.846–0.983) | 0.911 (0.850–0.961) | .37 |
| Pulmonary fibrotic sequela | 0.933 (0.893–0.973) | 0.911 (0.816–0.978) | 0.872 (0.766–0.958) | 0.891 (0.818–0.954) | .26 |
| Pleural effusion | 0.993 (0.980–1.000) | 0.950 (0.842–1.000) | 1.000 (1.000–1.000) | 0.974 (0.914–1.000) | .32 |
| Mosaic attenuation pattern | 1.000 (1.000–1.000) | 1.000 (1.000–1.000) | 1.000 (1.000–1.000) | 1.000 (1.000–1.000) | NA |
| Peribronchial thickening | 0.967 (0.933–0.993) | 0.864 (0.687–1.000) | 0.905 (0.762–1.000) | 0.884 (0.757–0.976) | .74 |
| Consolidation | 0.953 (0.913–0.980) | 0.815 (0.652–0.958) | 0.917 (0.782–1.000) | 0.863 (0.744–0.950) | .41 |
| Bronchiectasis | 0.960 (0.927–0.987) | 0.769 (0.600–0.923) | 1.000 (1.000–1.000) | 0.870 (0.750–0.960) | .08 |
| Interlobular septal thickening | 0.980 (0.953–1.000) | 0.700 (0.375–1.000) | 1.000 (1.000–1.000) | 0.824 (0.545–1.000) | .16 |

**Table S2.** Performance evaluation of CT-BERT-JPN using raw machine-translated reports as input across 18 different findings. The table shows the model's performance metrics, including Accuracy, Precision, Recall, F1 score, and AUC-ROC. Values in parentheses represent 95% confidence intervals.

| Findings | Accuracy | Precision | Recall | F1 | AUC-ROC |
| --- | --- | --- | --- | --- | --- |
| Medical material | 0.973 (0.947–0.993) | 0.812 (0.583–1.000) | 0.929 (0.750–1.000) | 0.867 (0.692–0.973) | 0.997 (0.991–1.000) |
| Arterial wall calcification | 0.993 (0.980–1.000) | 0.980 (0.933–1.000) | 1.000 (1.000–1.000) | 0.990 (0.966–1.000) | 1.000 (1.000–1.000) |
| Cardiomegaly | 1.000 (1.000–1.000) | 1.000 (1.000–1.000) | 1.000 (1.000–1.000) | 1.000 (1.000–1.000) | 1.000 (1.000–1.000) |
| Pericardial effusion | 0.993 (0.980–1.000) | 1.000 (1.000–1.000) | 0.917 (0.727–1.000) | 0.957 (0.842–1.000) | 1.000 (1.000–1.000) |
| Coronary artery wall calcification | 0.987 (0.967–1.000) | 0.978 (0.927–1.000) | 0.978 (0.925–1.000) | 0.978 (0.943–1.000) | 1.000 (0.999–1.000) |
| Hiatal hernia | 1.000 (1.000–1.000) | 1.000 (1.000–1.000) | 1.000 (1.000–1.000) | 1.000 (1.000–1.000) | 1.000 (1.000–1.000) |
| Lymphadenopathy | 0.993 (0.980–1.000) | 0.974 (0.909–1.000) | 1.000 (1.000–1.000) | 0.987 (0.952–1.000) | 1.000 (0.998–1.000) |
| Emphysema | 0.987 (0.967–1.000) | 0.968 (0.893–1.000) | 0.968 (0.889–1.000) | 0.968 (0.909–1.000) | 1.000 (0.998–1.000) |
| Atelectasis | 1.000 (1.000–1.000) | 1.000 (1.000–1.000) | 1.000 (1.000–1.000) | 1.000 (1.000–1.000) | 1.000 (1.000–1.000) |
| Lung nodule | 0.980 (0.960–1.000) | 1.000 (1.000–1.000) | 0.963 (0.921–1.000) | 0.981 (0.959–1.000) | 0.991 (0.976–1.000) |
| Lung opacity | 0.960 (0.927–0.987) | 0.945 (0.875–1.000) | 0.945 (0.879–1.000) | 0.945 (0.899–0.984) | 0.993 (0.984–0.999) |
| Pulmonary fibrotic sequela | 0.960 (0.927–0.987) | 0.918 (0.830–0.982) | 0.957 (0.898–1.000) | 0.938 (0.880–0.981) | 0.986 (0.968–1.000) |
| Pleural effusion | 0.993 (0.980–1.000) | 0.950 (0.842–1.000) | 1.000 (1.000–1.000) | 0.974 (0.914–1.000) | 0.998 (0.993–1.000) |
| Mosaic attenuation pattern | 1.000 (1.000–1.000) | 1.000 (1.000–1.000) | 1.000 (1.000–1.000) | 1.000 (1.000–1.000) | 1.000 (1.000–1.000) |
| Peribronchial thickening | 0.993 (0.980–1.000) | 1.000 (1.000–1.000) | 0.952 (0.842–1.000) | 0.976 (0.914–1.000) | 0.995 (0.981–1.000) |
| Consolidation | 0.973 (0.947–0.993) | 0.885 (0.750–1.000) | 0.958 (0.852–1.000) | 0.920 (0.828–0.986) | 0.992 (0.978–1.000) |
| Bronchiectasis | 0.973 (0.940–0.993) | 0.833 (0.667–0.962) | 1.000 (1.000–1.000) | 0.909 (0.800–0.980) | 0.996 (0.987–1.000) |
| Interlobular septal thickening | 1.000 (1.000–1.000) | 1.000 (1.000–1.000) | 1.000 (1.000–1.000) | 1.000 (1.000–1.000) | 1.000 (1.000–1.000) |

**Figure S1.** Machine translation prompts for CT-RATE-JPN creation. The system prompt used in the study, instructing GPT-4o mini to act as a Japanese radiologist and translate radiology report findings from English to Japanese, with specific output formatting requirements.


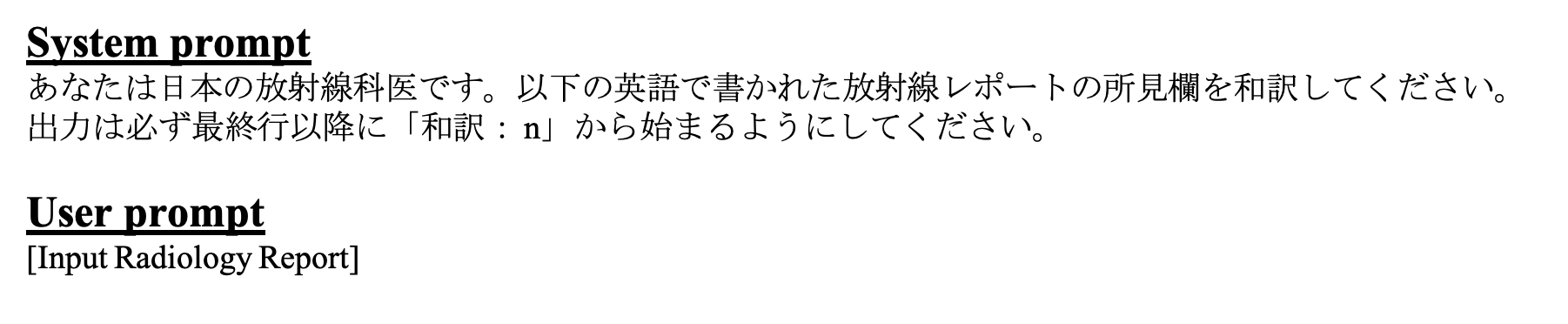


**Figure S2.** English translation of the machine translation prompts. The English version of the prompts shown in Supplementary Figure 1, provided to ensure reproducibility of the translation process.


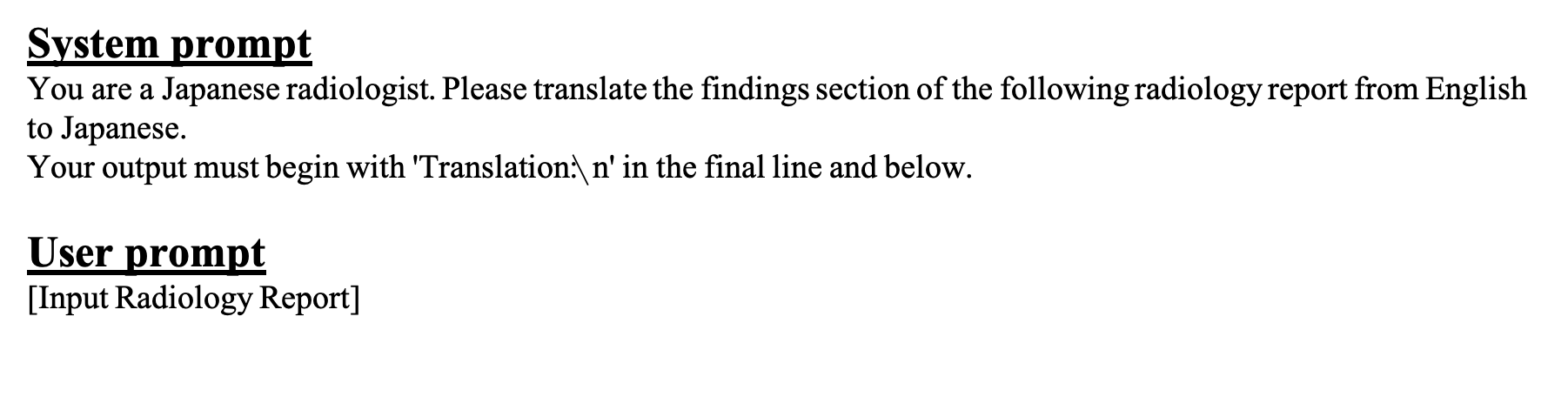


**Figure S3.** System and user prompts used for GPT-4o structured labeling in Japanese. The system prompt defines the AI's role in extracting structured labels from Japanese radiology reports, with detailed instructions for binary classification (0/1) of 18 specific CT findings and output format specifications. The user prompt requests structured label extraction from an input radiology report.
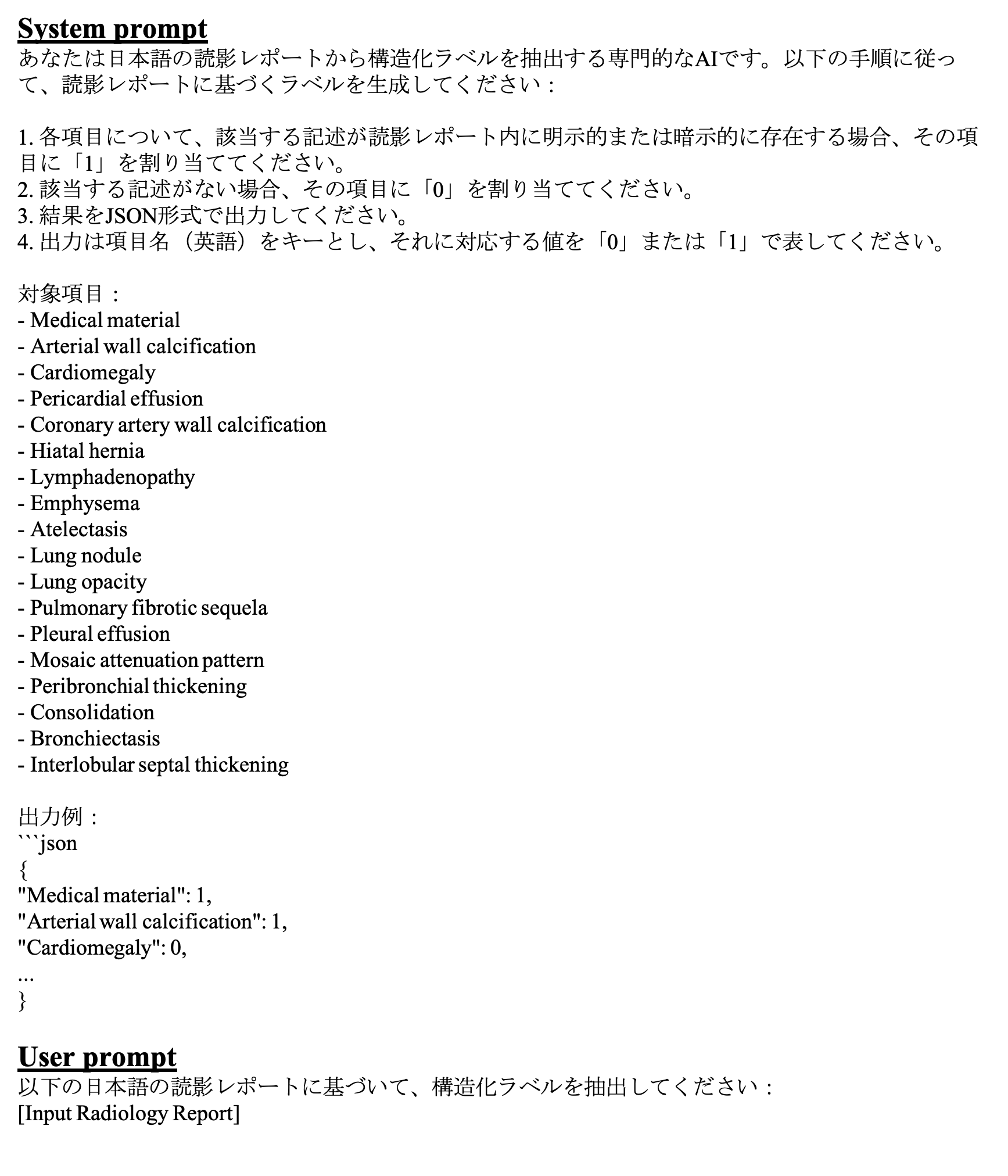


**Figure S4.** English translation of the system and user prompts used for GPT-4o structured labeling. This figure shows the complete English version of the prompts presented in Supplementary Figure 3, including the system prompt detailing the AI's role and instructions for binary classification of 18 CT findings, and the user prompt requesting structured label extraction from an input radiology report.


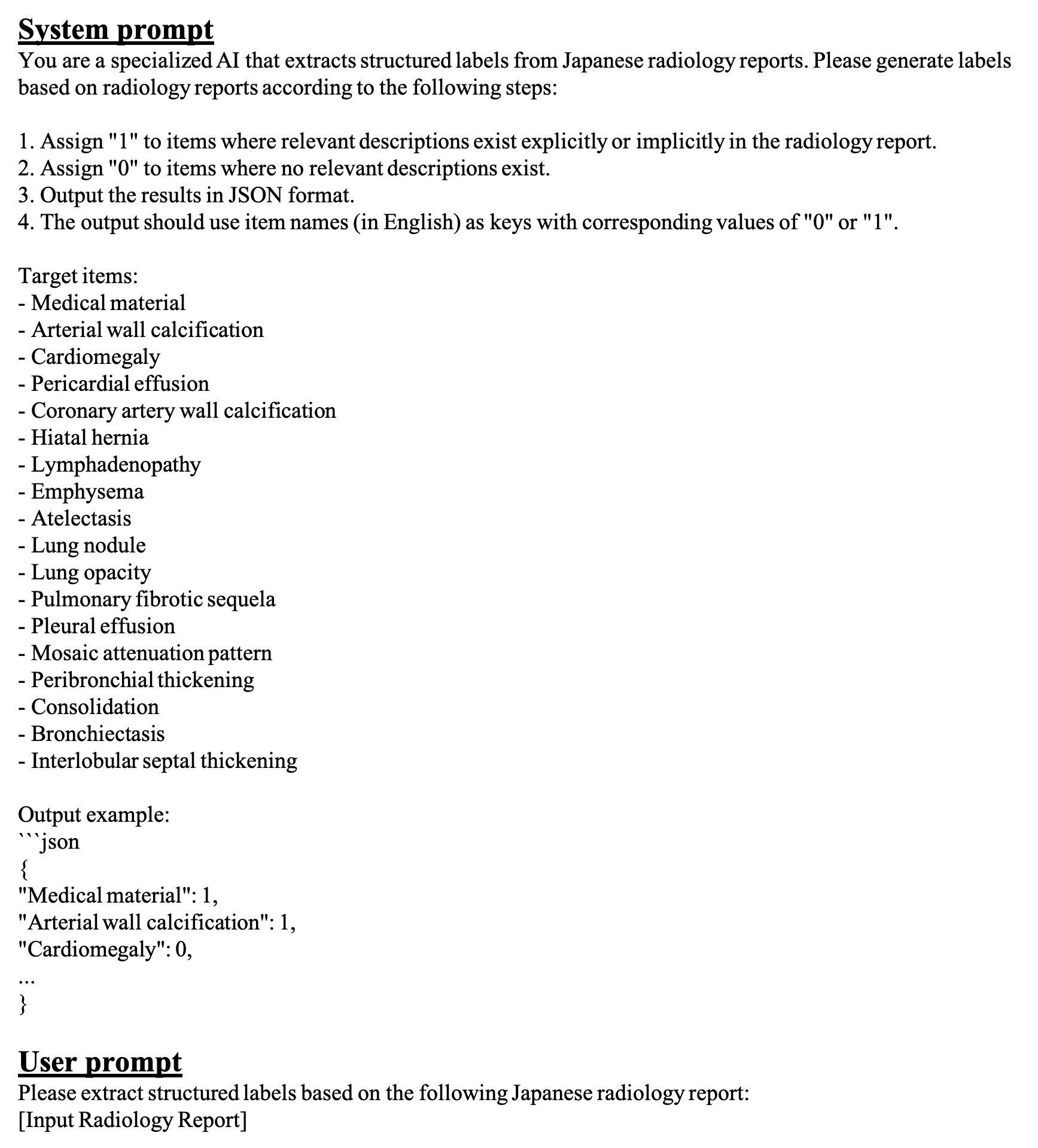


**Figure S5.** Original English radiology report referenced in Figure 6.


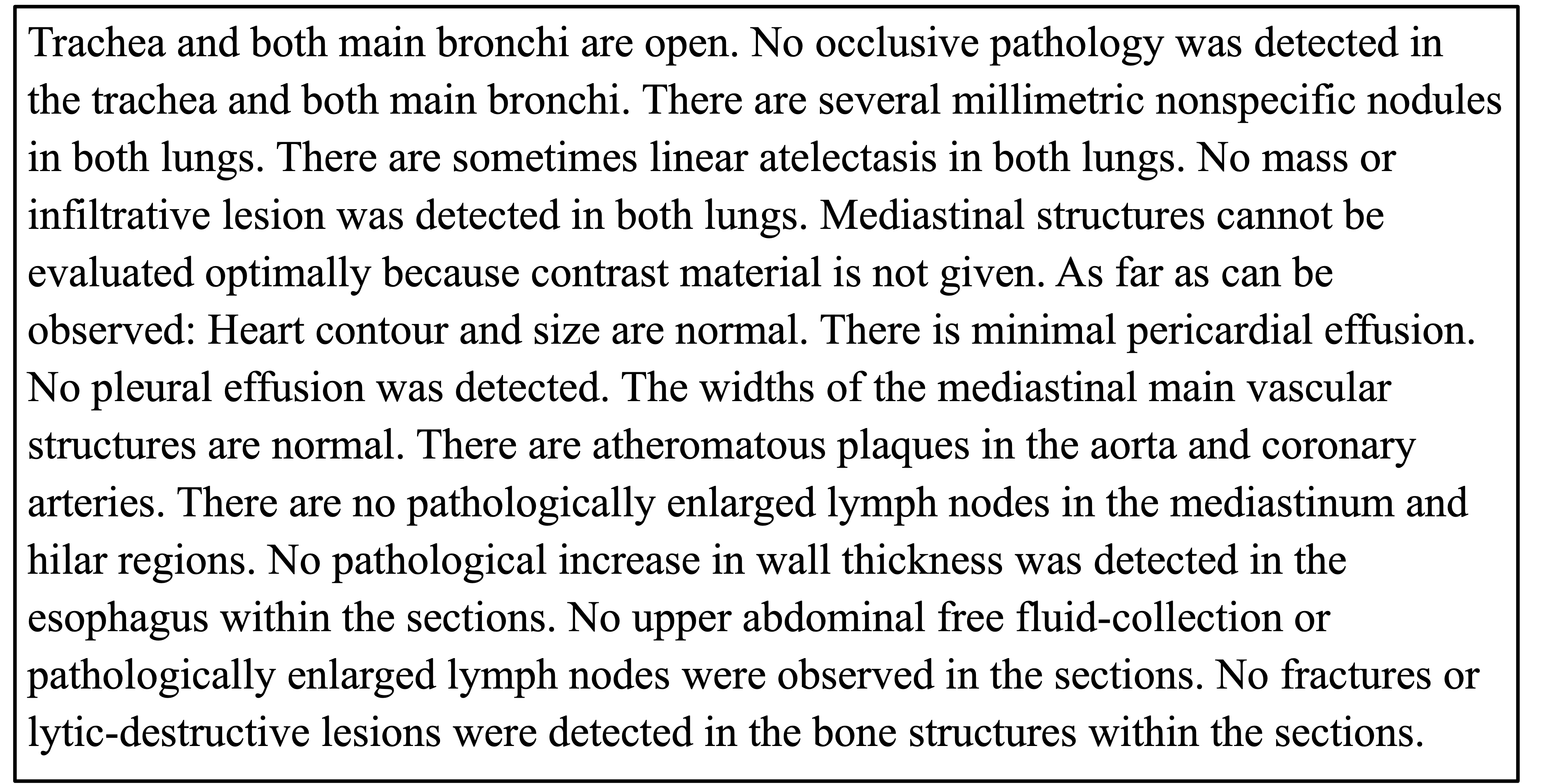

Supplement: Multimedia Appendix 1 [file medinform-v13-e71137-s001.docx]
